# Supplementary material for: COZOID: contact zone identifier for visual analysis of protein-protein interactions
Source: BMC Bioinformatics. 2018 Apr 6;19:125. doi: 10.1186/s12859-018-2113-6 (PMC5889581; doi:10.1186/s12859-018-2113-6)
Supplement: Supplementary file 2 — Software build. Executable binary file of the software tool. (ZIP 118,784 kb) [file 12859_2018_2113_MOESM2_ESM.zip › cozoid/java/docs/beansbinding-1.2.1-doc.zip!/index-files/index-18.html]

R-Index (Beans Binding)


---


|  |  |  |
| --- | --- | --- |
| **Overview** \| Package \| Class \| Use \| **Tree** \| **Deprecated** \| **Index** \| **Help** | |  |
| **PREV LETTER**   **NEXT LETTER** | **FRAMES**    **NO FRAMES**     **All Classes** |  |


A B C D E F G H I J K L M N O P Q R S T U V W 

---


## **R**

**RBRACK** - Static variable in interface org.jdesktop.el.impl.parser.ELParserConstants: **readChar()** - Method in class org.jdesktop.el.impl.parser.SimpleCharStream: **readExternal(ObjectInput)** - Method in class org.jdesktop.el.impl.lang.FunctionMapperImpl.Function: **readExternal(ObjectInput)** - Method in class org.jdesktop.el.impl.lang.FunctionMapperImpl: **readExternal(ObjectInput)** - Method in class org.jdesktop.el.impl.lang.VariableMapperImpl: **readExternal(ObjectInput)** - Method in class org.jdesktop.el.impl.MethodExpressionImpl: **readExternal(ObjectInput)** - Method in class org.jdesktop.el.impl.MethodExpressionLiteral: **readExternal(ObjectInput)** - Method in class org.jdesktop.el.impl.ValueExpressionImpl: **readExternal(ObjectInput)** - Method in class org.jdesktop.el.impl.ValueExpressionLiteral: **ReflectionUtil** - Class in org.jdesktop.el.impl.util: Utilities for Managing Serialization and Reflection **refresh()** - Method in class org.jdesktop.beansbinding.Binding: Fetches the value of the source property for the source object and sets it as the value of the target property for the target object. **refreshAndNotify()** - Method in class org.jdesktop.beansbinding.Binding: The same as `Binding.refresh()` with the additional behavior of notifying all registered `BindingListeners` with `synced` if `refresh` returns `null` or `syncFailed` if `refresh` returns a `SyncFailure`. **refreshAndNotifyUnmanaged()** - Method in class org.jdesktop.beansbinding.Binding: A protected version of `Binding.refreshAndNotify()` that allows managed subclasses to refresh and notify without throwing an exception for being managed. **refreshUnmanaged()** - Method in class org.jdesktop.beansbinding.Binding: A protected version of `Binding.refresh()` that allows managed subclasses to refresh without throwing an exception for being managed. **ReInit(InputStream)** - Method in class org.jdesktop.el.impl.parser.ELParser: **ReInit(Reader)** - Method in class org.jdesktop.el.impl.parser.ELParser: **ReInit(ELParserTokenManager)** - Method in class org.jdesktop.el.impl.parser.ELParser: **ReInit(SimpleCharStream)** - Method in class org.jdesktop.el.impl.parser.ELParserTokenManager: **ReInit(SimpleCharStream, int)** - Method in class org.jdesktop.el.impl.parser.ELParserTokenManager: **ReInit(Reader, int, int, int)** - Method in class org.jdesktop.el.impl.parser.SimpleCharStream: **ReInit(Reader, int, int)** - Method in class org.jdesktop.el.impl.parser.SimpleCharStream: **ReInit(Reader)** - Method in class org.jdesktop.el.impl.parser.SimpleCharStream: **ReInit(InputStream, int, int, int)** - Method in class org.jdesktop.el.impl.parser.SimpleCharStream: **ReInit(InputStream)** - Method in class org.jdesktop.el.impl.parser.SimpleCharStream: **ReInit(InputStream, int, int)** - Method in class org.jdesktop.el.impl.parser.SimpleCharStream: **removeBinding(Binding)** - Method in class org.jdesktop.beansbinding.BindingGroup: Removes a `Binding` from this group. **removeBindingListener(BindingListener)** - Method in class org.jdesktop.beansbinding.Binding: Removes a `BindingListener` from the `Binding`. **removeBindingListener(BindingListener)** - Method in class org.jdesktop.beansbinding.BindingGroup: Removes a `BindingListener` from the group. **removeColumnBinding(JTableBinding<E, SS, TS>.ColumnBinding)** - Method in class org.jdesktop.swingbinding.JTableBinding: Removes the given `ColumnBinding` from the list maintained by this `JTableBinding`. **removeColumnBinding(int)** - Method in class org.jdesktop.swingbinding.JTableBinding: Removes the `ColumnBinding` with the given index from the list maintained by this `JTableBinding`. **removed(int, int)** - Method in class org.jdesktop.swingbinding.impl.ListBindingManager: **removeObservableListListener(ObservableListListener)** - Method in interface org.jdesktop.observablecollections.ObservableList: Removes a listener. **removeObservableMapListener(ObservableMapListener)** - Method in interface org.jdesktop.observablecollections.ObservableMap: Removes a listener from this observable map. **removePropertyChangeListener(PropertyChangeListener)** - Method in class org.jdesktop.beansbinding.Binding: Removes a `PropertyChangeListener` from the `Binding`. **removePropertyChangeListener(String, PropertyChangeListener)** - Method in class org.jdesktop.beansbinding.Binding: Removes a `PropertyChangeListener` from the `Binding` for the given property name. **removePropertyChangeListener(PropertyChangeListener)** - Method in class org.jdesktop.swingbinding.adapters.BeanAdapterBase: **removePropertyChangeListener(String, PropertyChangeListener)** - Method in class org.jdesktop.swingbinding.adapters.BeanAdapterBase: **removePropertyStateListener(S, PropertyStateListener)** - Method in class org.jdesktop.beansbinding.ObjectProperty: Does nothing; the state of an `ObjectProperty` never changes so listeners aren't useful. **removePropertyStateListener(S, PropertyStateListener)** - Method in class org.jdesktop.beansbinding.Property: Removes a `PropertyStateListener` for the given source. **removePropertyStateListener(S, PropertyStateListener)** - Method in class org.jdesktop.beansbinding.PropertyHelper: Removes a `PropertyStateListener` for the given source. **RESOLVABLE\_AT\_DESIGN\_TIME** - Static variable in class org.jdesktop.el.ELResolver: The attribute name of the named attribute in the `FeatureDescriptor` that specifies whether the variable or property can be resolved at runtime. **resolvedIdentifier(Object, Object)** - Method in class org.jdesktop.el.impl.lang.EvaluationContext: **resolvedProperty(Object, Object)** - Method in class org.jdesktop.el.impl.lang.EvaluationContext: **resolveFunction(String, String)** - Method in class org.jdesktop.el.FunctionMapper: Resolves the specified prefix and local name into a `java.lang.Method`. **resolveFunction(String, String)** - Method in class org.jdesktop.el.impl.lang.FunctionMapperFactory: **resolveFunction(String, String)** - Method in class org.jdesktop.el.impl.lang.FunctionMapperImpl: **resolveVariable(String)** - Method in class org.jdesktop.el.impl.lang.VariableMapperFactory: **resolveVariable(String)** - Method in class org.jdesktop.el.impl.lang.VariableMapperImpl: **resolveVariable(String)** - Method in class org.jdesktop.el.VariableMapper: **ResourceBundleELResolver** - Class in org.jdesktop.el: Defines property resolution behavior on instances of `ResourceBundle`. **ResourceBundleELResolver()** - Constructor for class org.jdesktop.el.ResourceBundleELResolver: **RPAREN** - Static variable in interface org.jdesktop.el.impl.parser.ELParserConstants

---


|  |  |  |
| --- | --- | --- |
| **Overview** \| Package \| Class \| Use \| **Tree** \| **Deprecated** \| **Index** \| **Help** | |  |
| **PREV LETTER**   **NEXT LETTER** | **FRAMES**    **NO FRAMES**     **All Classes** |  |


A B C D E F G H I J K L M N O P Q R S T U V W 

---
